# The prevalence of histologic acute chorioamnionitis among HIV infected pregnant women in Uganda and its association with adverse birth outcomes

**DOI:** 10.1371/journal.pone.0215058

**Published:** 2019-04-11

**Authors:** John Ategeka, Razack Wasswa, Peter Olwoch, Abel Kakuru, Paul Natureeba, Atis Muehlenbachs, Moses R. Kamya, Grant Dorsey, Gabrielle Rizzuto

**Affiliations:** 1 Infectious Diseases Research Collaboration, Kampala, Uganda; 2 Division of High-Consequence Pathogens and Pathology, National Center for Emergin and Zoonotic Infectious Diseases, Centers for Disease Control and Prevention, Atlanta, Georgia, United States of America; 3 School of Medicine, Makerere University College of Health Sciences, Kampala, Uganda; 4 Department of Medicine, Division of Infectious Diseases, University of California San Francisco, San Francisco, California, United States of America; 5 Department of Anatomic Pathology, University of California San Francisco, San Francisco, California, United States of America; Duke University, UNITED STATES

## Abstract

**Background:**

Preterm birth (PTB) is a leading cause of neonatal mortality and longer-term morbidity. Acute chorioamnionitis (ACA) is a common cause of PTB, however, there are limited data on the prevalence of ACA and its association with PTB in resource limited settings.

**Methods:**

Data and samples came from a clinical trial evaluating novel strategies for the prevention of malaria in HIV infected pregnant women in Uganda. Women were enrolled between 12–28 weeks of gestation and followed through delivery. For each placenta delivered, three placental tissue types (membrane roll, umbilical cord and chorionic plate/villous parenchyma) were collected. Slides were assessed for diagnosis of maternal and fetal ACA by microscopic evaluation of neutrophilic infiltration using a standardized grading scale. The primary outcomes were PTB (<37 weeks), low birth weight (LBW, <2500 grams), and small-for-gestational age (SGA, birth weight <10^th^ percentile for age). Univariate and multivariate logistic regression were used to estimate associations between 1) maternal characteristics (age, education, wealth, gravidity, gestational age at enrollment, placental malaria, anti-malarial prophylaxis treatment regimen, HIV disease parameters) and ACA, and 2) associations between ACA and adverse birth outcomes.

**Findings:**

A total of 193 placentas were included in the analysis. The prevalence of maternal and fetal ACA was 44.5% and 28.0%, respectively. HIV infected women between 28–43 years of age had a higher risk of maternal ACA compared to those between 17–21 years of age (50.9% vs. 19.1%; aOR = 4.00 (1.10–14.5), *p* = 0.04) and the diagnosis of severe maternal ACA was associated with a significantly higher risk of PTB (28.6% vs. 6.0%; aOR = 6.04 (1.87–19.5), *p* = 0.003), LBW (33.3% vs. 9.4%; aOR = 4.86 (1.65–14.3); *p* = 0.004), and SGA (28.6% vs. 10.1%; aOR = 3.70 (1.20–11.4), *p* = 0.02). No maternal characteristics were significantly associated with fetal ACA and the diagnosis of fetal ACA was not associated with adverse birth outcomes.

**Conclusions:**

Histological evidence of severe maternal ACA was associated with an increased risk of PTB, LBW, and SGA in HIV infected, pregnant Ugandan women.

## Introduction

Preterm birth (PTB, delivery occurring prior to 37 weeks) and low birth weight (LBW, weight <2500 grams) are major determinants of infant mortality and morbidity during childhood [1, 2]. PTB is a leading cause of death among neonates [3], complications arising from PTB are the leading cause of mortality among children less than five years of age worldwide [4], and PTB can result in long term neurodevelopmental difficulties for surviving children [5]. PTB is often associated with LBW, and LBW is an important stand-alone risk factor for infant death [3]. Rates of PTB range from 5–18% of live neonates [5], with the highest incidences reported in sub-Saharan Africa and South Asia [3, 5]. The United States Agency for International Development (USAID) cooperative agreement project to combat PTB and LBW in Africa and Asia recently estimated that, in Uganda, approximately 226,000 babies are born preterm every year and 12,500 deaths occur as a direct consequence of prematurity [6].

While the pathologic trigger for the majority of PTB cases is unknown, the most commonly identified trigger of PTB is acute chorioamnionitis (ACA) [7, 8]. ACA is defined as the maternal and/or fetal inflammatory response to microbial infection of the amniotic sac [9, 10]. Most ACA cases are caused by bacterial or fungal organisms that ascend to the placenta from the lower genital tract, while a minority of infections occur secondary to seeding of the uterus from the maternal bloodstream or from direct abdominal extension [11]. Obstetricians diagnose “clinical” ACA when characteristic maternal symptoms, such as fever and tenderness, are present [11]. However, some infections may progress sub-clinically [12]. Therefore, gold standard diagnosis is made during microscopic examination of the placenta, and this has often been referred to as “histologic” ACA [9, 10]. In response to infection, maternal and fetal acute inflammatory cells (maternal and fetal neutrophils, and fetal eosinophils) exit vasculature to infiltrate the placental membranes (chorioamnion), umbilical cord, and chorionic plate. The neutrophil response is such that there is stereotyped progression of neutrophil and eosinophil location and cell density [10], and there is both high accuracy and inter-observer concordance for diagnosing the presence of inflammation and grading the severity [9].

It is possible that the susceptibility to ACA and the pathogenesis of placental infection in Uganda might be altered by the presence of other infections endemic to this location, notably HIV and malaria. According to the joint United Nations Programme on HIV/AIDS (UNAIDS), greater than 600,000 Ugandan women are HIV seropositive [13]. Despite the benefits of antiretroviral medication in pregnancy, some evidence suggests a moderate association of protease inhibitor treatment with increased PTB [14], and HIV status itself may be a risk factor for PTB [15]. We recently published a study that showed an association between placental malaria (PM) and poor birth outcomes (PTB and LBW) in the same Ugandan cohort analyzed here [16], and Abrams et al found that both HIV seropositivity and histologic ACA were risk factors for PTB in malaria-exposed women in Malawi [17]. While some evidence from Malawi links PM to PTB [18], another study from the same country did not observe such an association [17]. Thus, the correlation between these infections, treatment regimens, and poor birth outcomes has not been conclusively studied.

Further, little is known about the prevalence of ACA and associated risk factors in Uganda. A single study in South Western Uganda identified histologic ACA in 34.1% of women delivering at full term gestation [19]. Unfortunately, this study did not examine placentas from pre-term deliveries, and microscopic evaluation was limited to the placental membranes and did not include the umbilical cord and chorionic plate, two locations that are important to examine in order to avoid false negative diagnosis. To better understand the epidemiology of ACA in Uganda, we undertook a study to assess the prevalence of histologic ACA in HIV infected pregnant Ugandan women and to evaluate the association between ACA and the risk of adverse birth outcomes (PTB, LBW, and SGA). Although not all known risk factors for ACA and adverse outcomes were assessed, several important maternal factors associated with ACA were examined. Such assessments are important for improving obstetrical practice policies and preventive measures in Uganda and similar resource-limited settings.

## Materials and methods

### Study design, site and participant population

Clinical data and biological samples for this study came from a double-blinded randomized clinical trial (Registration number NCT02282293) evaluating intermittent preventive therapy for malaria in pregnancy (IPTp) among HIV infected women in Tororo, Uganda [20]. Two hundred HIV infected pregnant women at least 16 years of age were enrolled at 12–28 weeks gestational age, between December 2014 to November 2015, and were randomized to daily trimethoprim-sulfamethoxazole (TMP-SMX) plus monthly dihydroartemisinin-piperaquine (DP) or daily TMP-SMX alone [20]. The study physician estimated gestational age using last menstrual period and fetal measurements obtained by a standardized abdominal ultrasound exam. All women were provided with combination antiretroviral therapy (ART) consisting of Efavirenz (EFV)/tenofovir/lamivudine. Women received routine HIV care per the Uganda Ministry of Health guidelines, and HIV-1 RNA monitoring was additionally performed. Participants were prospectively followed until delivery, at which point the placenta was harvested and prepared (as described below) for microscopic examination and diagnosis of ACA.

### Study procedures and follow up

At enrollment, a long-lasting insecticide treated net was provided to every participant and a detailed history including a household questionnaire and physical assessment/examination was obtained. Study participants were closely monitored throughout pregnancy by monthly assessments and collection of blood samples, and all their health care needs were provided by a dedicated study clinic open seven days a week. Participants were encouraged to deliver at the hospital. However, in the event of a home or other health facility delivery, participants were visited by the study staff at the time of delivery or as soon as possible afterwards.

Upon delivery, a comprehensive assessment of birth outcomes was made, including neonatal evaluation for congenital anomalies, measurement of birth weight and gestational age. All placental sampling was done within 30 minutes of delivery. Placental specimens were fixed in 10% Neutral Buffered Formalin for 24 hours, and stored at room temperature in 70% ethanol prior to tissue processing. The following placental tissues were collected: placental membranes (“membrane roll”), umbilical cord (two cross-sectional slices, one proximal and one distal to where the cord inserts into the placental disc), chorionic plate with villous parenchyma, and basal plate with villous parenchyma. The basal plate/villous parenchyma section was used for diagnosis of PM in our separately reported studies [16, 20, 21], while the other three sections (membrane roll, umbilical cord, chorionic plate/villous parenchyma) were used as described below for the diagnosis of ACA in the study reported here.

### Laboratory methods

For microscopic diagnosis of ACA, three placental specimens were utilized: membrane roll, umbilical cord, and chorionic plate/villous parenchyma. In the Tororo, Uganda laboratory, specimens were dehydrated through a series of ethanol washes, cleared in xylene and embedded in paraffin wax blocks. A 3 μm thick section from each tissue block was obtained using a rotary microtome and sections mounted onto glass slides via a floatation water bath. Slides were baked in a hot air oven at 60°C for 30 minutes, de-paraffinized in xylene, dehydrated through a series of ethanol washes, stained with Hematoxylin and Eosin (H&E), and mounted with organic mounting media.

**Fig 1:**
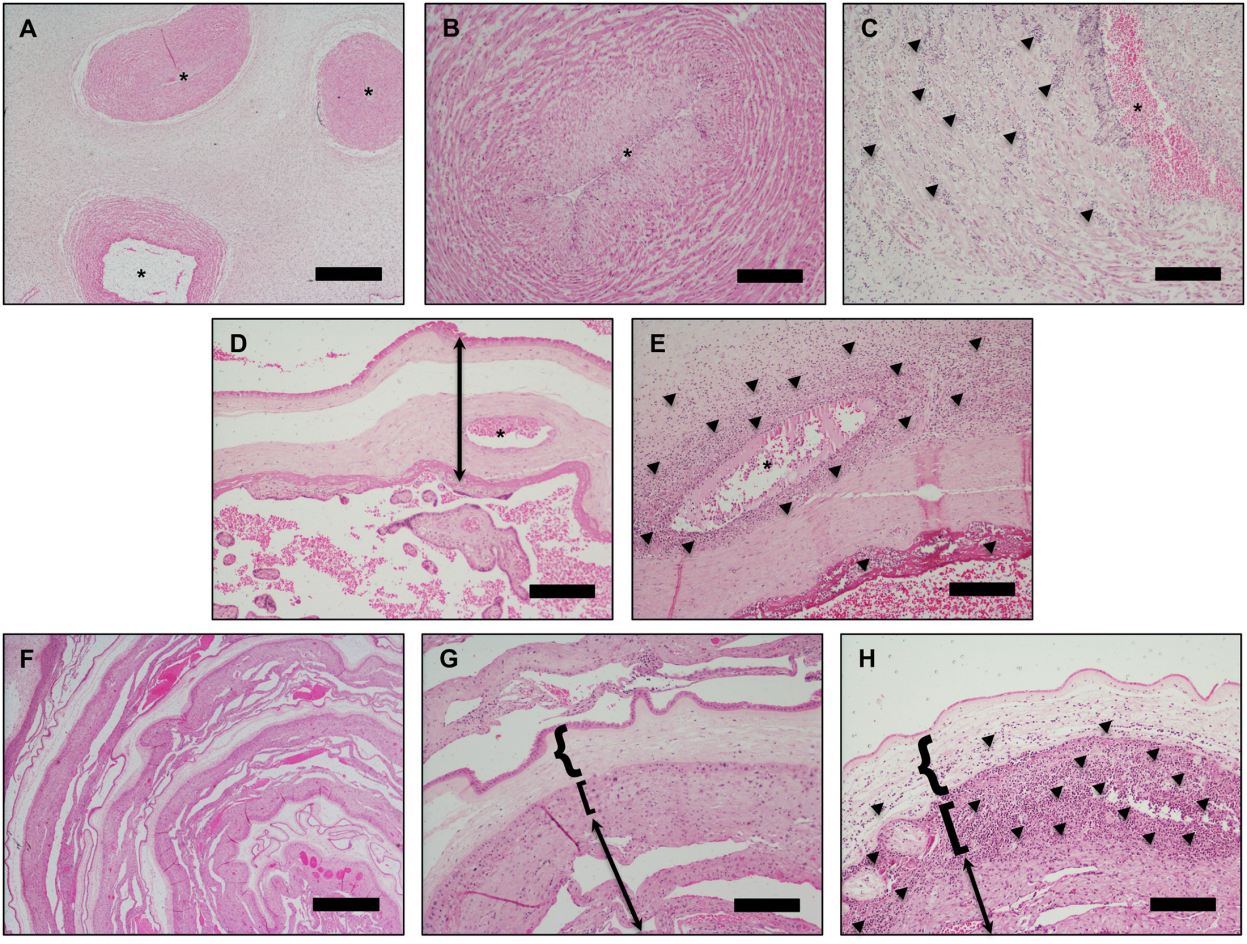
Representative placental histology. **(A&B)** Normal umbilical cord. *indicates lumen of umbilical vessels. **(C)** Umbilical vein with heavy neutrophilic infiltrate (arrows point to aggregates of neutrophils) and early degeneration of smooth muscle cells diagnostic of severe fetal ACA. **(D)** Normal chorionic plate/villous parenchyma. Arrow indicates the chorionic plate, and * indicates lumen of a fetal chorionic plate vessel. **(E)** Chorionic plate with heavy neutrophilic infiltrate in the walls of a fetal chorionic plate vessel (severe fetal ACA) and neutrophils in the subchorionic fibrin layer (maternal ACA). Arrows point to aggregates of neutrophils. **(F&G)** Normal membrane roll. **(H)** Membrane roll with neutrophilic microabscess diagnostic of severe maternal ACA (arrows point to neutrophils). In G and H, **{**-bracket indicates amnion, **[**-bracket indicates chorion, and arrow indicates decidua parietalis. Scale bars are 1 mm (panels A, D, F) and 200 μM (panels B, C, E, G, H).

Diagnosis of ACA was made by standard light microscopic examination (Nikon Eclipse Ci-L microscope with DS-Ri2 color camera) of the three placental biopsy slides (membrane roll, umbilical cord, and chorionic plate/villous parenchyma) (Fig 1). Diagnosis of a maternal acute inflammatory response to infection (“maternal ACA”) was indicated by detection of neutrophils within the chorioamnion layers of the membrane roll and/or the chorionic plate. Diagnosis of a fetal acute inflammatory response to infection (“fetal ACA”) was indicated by detection of neutrophils with or without eosinophils within the vascular smooth muscle of the umbilical cord vessels and/or the chorionic plate vasculature [9]. Diagnosis was rendered in blinded fashion by a placental pathologist (G.R.) and noted on a standardized case record form.

Grading of maternal ACA and fetal ACA was assigned using the standardized diagnostic framework of the Perinatal Section of the Society for Pediatric Pathologists [9]. Grade was recorded on the case record form as either: no evidence of ACA, mild (scattered mostly single, isolated neutrophils), moderate (small clusters of neutrophils) and severe (presence of neutrophilic microabscesses). Specifically, severe maternal ACA was defined as the presence of microabscesses measuring greater than or equal to 10 x 20 neutrophils in extent, and present within at least three foci, or with neutrophils present as a contiguous band. Severe fetal ACA was defined as the presence of near confluent intramural neutrophils with attenuation/degeneration of vascular smooth muscle cells.

### Variables of interest

The maternal characteristics examined included: maternal age and gestational age at enrollment, education, household wealth index, and gravity. Maternal characteristics pertaining to malaria included: IPTp regimen (either TMP-SMX plus DP or TMP-SMX alone), and evidence of PM defined by histopathology. Additionally, the following maternal characteristic pertaining to HIV infection were assessed: duration since HIV diagnosed, duration since ART was begun, WHO HIV stage at enrollment, CD4 T-cell count at delivery, and viral load at delivery. None of the women enrolled were WHO HIV stage 4 (defined as AIDS). ACA was categorized as maternal ACA (the presence of maternal acute inflammation, defined above) and fetal ACA (the presence of fetal acute inflammation, defined above). Maternal and fetal ACA were each categorized into a grading scale: none/mild, moderate and severe (defined above). The birth outcomes assessed were PTB (delivery at <37 weeks), LBW (birth weight <2500 grams, and SGA (birth weight <10^th^ percentile for age).

### Statistical analysis

Data were coded, double entered into a Microsoft access database and analyzed using Stata 14 (Stata Corp, College Station TX). Participants’ baseline maternal characteristics were expressed as means ± SD for continuous variables, while categorical variables were expressed as proportions. Univariate and multivariate logistic regression were used to 1) measure associations between maternal characteristics and maternal and fetal ACA, and 2) measure associations between maternal and fetal ACA and the risk of adverse birth outcomes. *P* values < 0.05 were considered statistically significant. Measures of association in both univariate and multivariate models were expressed as odds ratios (OR) or adjusted odds ratios (aOR) and respective 95% confidence intervals (CI). Gravidity was not included in the final multivariate model due to correlation with maternal age.

### Ethical approval

All study participants provided informed written consent. Ethical approval was obtained from the Uganda National Council of Science and Technology (UNCST #HS 1708), Makerere University School of Medicine Research and Ethics Committee, the Makerere University School of Biomedical Sciences Research and Ethics Committee (#SBS-REC 159), and the University of California, San Francisco, Committee on Human Research (CHR #14–13900).

## Results

### Maternal characteristics of study participants

**Fig 2:**
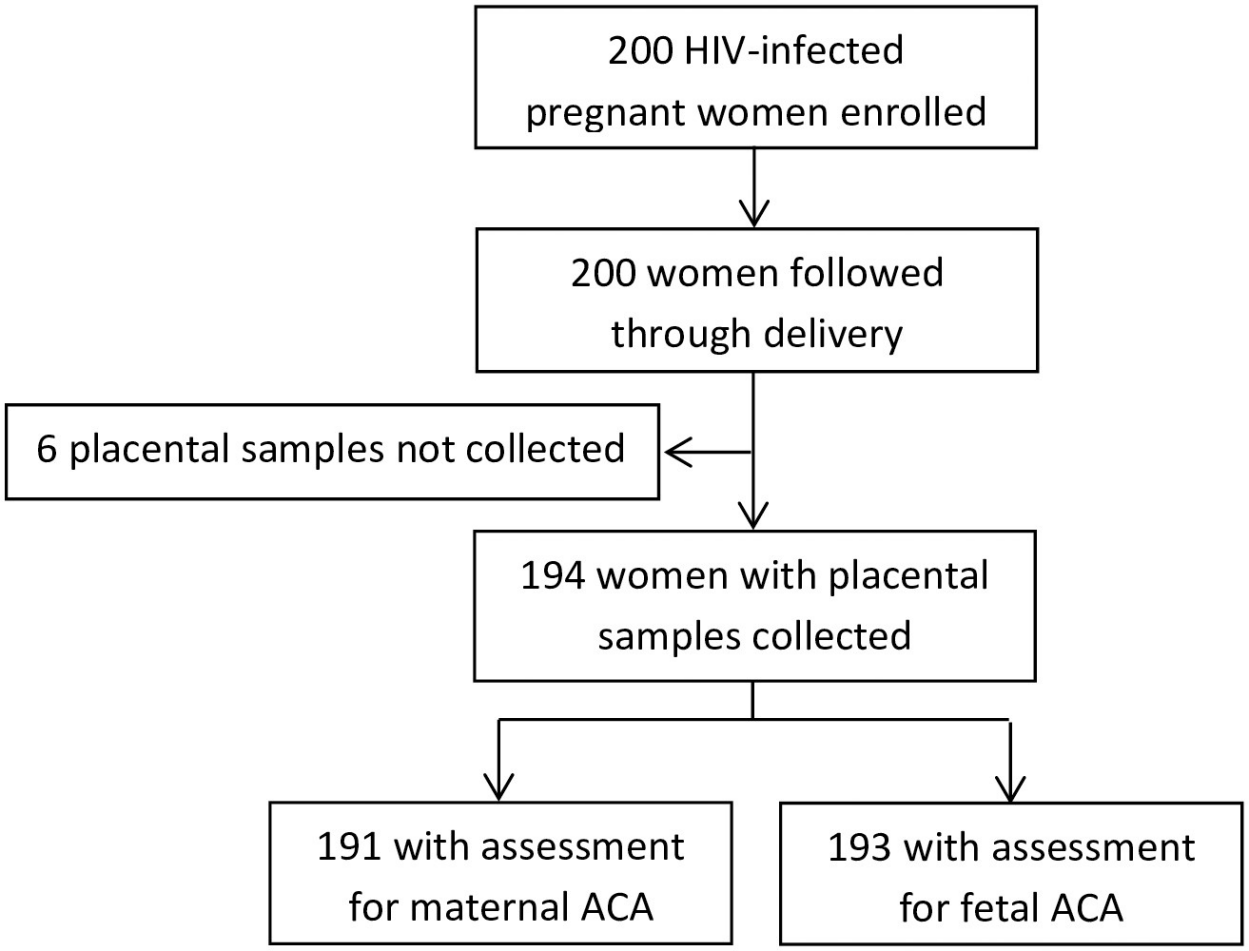
Participant trial profile. Summary of placental samples acquired.

A total of 200 HIV infected pregnant women were enrolled and followed through delivery, and 194 placenta samples were collected. A total of 191 and 193 placental samples were successfully assessed for maternal and fetal ACA, respectively, and included in the analysis as shown in Fig 2. A majority of the women were 28–43 years old age (61.7%), completed primary level of education (55.4%), and were multigravida (78.8%), while only 4.7% had evidence of PM diagnosed by histopathology (Table 1). At enrollment, most women had been on combination ART for >90 days (67.4%), and were WHO HIV disease stage 1 (asymptomatic) (94.8%). At delivery, a majority of the women showed a normal CD4 T-cell count (61.7%), and only 23.8% had detectable HIV virus.

**Table 1:** Maternal characteristics among samples with any assessment for ACA.

Characteristic	Prevalence
**Age categories at enrollment**	
17–21 years	21 (10.9%)
22–27 years	53 (27.5%)
28–43 years	119 (61.7%)
**Highest level of formal education**	
None	43 (22.3%)
Primary school	107 (55.4%)
Secondary school	25 (13.0%)
Beyond secondary school	18 (9.3%)
**Household wealth index**	
Lowest tertile	64 (33.2%)
Middle tertile	62 (32.1%)
Highest tertile	67 (34.7%)
**Gravidity**	
Primigravida	18 (9.3%)
Secundigravida	23 (11.9%)
Multigravida	152 (78.8%)
**Gestational age at enrollment**	
12–16 weeks	51 (26.4%)
16–20 weeks	53 (27.5%)
20–24 weeks	54 (28.0%)
24–28 weeks	35 (18.1%)
**Duration since HIV first diagnosed at enrollment**	
>5 years	62 (32.2%)
2–5 years	55 (28.5%)
6 months–2 years	37 (19.2%)
≤ 6 months	39 (20.2%)
**Duration on ART at enrollment**	
>90 days	130 (67.4%)
1–90 days	25 (13.0%)
Started on ART at time of enrollment	38 (19.7%)
**WHO HIV disease stage 2 or 3 at enrollment**	10 (5.2%)
**IPTp regimen during pregnancy**	
Daily TMP-SMX	95 (49.2%)
Daily TMP-SMX + monthly DP	98 (50.8%)
**CD4 T cell count at delivery**	
>500 cells/mm^3^	119 (61.7%)
250–500 cell/mm^3^	58 (30.1%)
<250 cells/mm^3^	16 (8.3%)
**Detectable HIV viral load at delivery**	46 (23.8%)
**Placental malaria by histopathology at delivery**	9 (4.7%)

### Prevalence of ACA in HIV infected pregnant women

Overall, microscopic exam revealed that 102/193 (52.8%) of women were diagnosed with either maternal and/or fetal ACA. Maternal ACA was diagnosed in 44.5% of samples (22.5% mild, 11.0% moderate and 11.0% severe), while fetal ACA was observed in 27.9% of the samples (17.6% mild, 9.3% moderate and 1.0% severe).

### Associations between maternal characteristics and ACA

Associations between maternal characteristics and the diagnosis of maternal ACA are shown in Table 2. Only one statistically significant association was identified in multivariate analysis. Older age at enrollment was associated with increased odds of maternal ACA, with women 28–43 years of age having ~4 times the odds of maternal ACA compared to those 17–21 years of age in multivariate analysis (aOR = 4.00 (1.10–14.5), *p* = 0.04). Regarding the two different IPTp regimens, there was a lower prevalence of maternal ACA in women maintained on TMP-SMX plus DP versus TMP-SMX alone, but this association was of borderline statistical significance (aOR = 0.57 (0.31–1.06), *p* = 0.07).

**Table 2:** Associations between maternal characteristics and any evidence of maternal ACA.

Characteristic	Category	Risk of any maternal ACA	Univariate		Multivariate	
			OR (95% CI)	p-value	aOR (95% CI)	p-value
Age in years at enrollment	17–21	4/21 (19.1%)	reference		reference	
	22–27	21/52 (40.4%)	2.88 (0.85–9.77)	0.09	2.64 (0.71–9.86)	0.15
	28–43	60/118 (50.9%)	4.40 (1.40–13.9)	0.01	4.00 (1.10–14.5)	0.04
Formal education	Any	63/148 (42.6%)	reference		reference	
	None	22/43 (51.2%)	1.41 (0.72–2.79)	0.32	1.15 (0.53–2.51)	0.73
Household wealth index	Lowest third	25/63 (39.7%)	reference		reference	
	Middle third	28/61 (45.9%)	1.29 (0.63–2.63)	0.48	1.19 (0.55–2.56)	0.66
	Highest third	32/67 (47.8%)	1.39 (0.69–2.79)	0.35	1.53 (0.70–3.35)	0.29
Gravidity	Multigravida	69/151 (46.4%)	reference		Not included in final model due to correlation with maternal age	
	Secundigravida	9/22 (40.9%)	0.82 (0.33–2.04)	0.67		
	Primigravida	7/18 (38.9%)	0.76 (0.28–2.06)	0.58		
Gestational age at enrollment	> 20–28 weeks	34/88 (38.6%)	reference		reference	
	> 16–20 weeks	25/53 (47.2%)	1.42 (0.71–2.82)	0.32	1.23 (0.58–2.58)	0.59
	12–16 weeks	26/50 (52.0%)	1.72 (0.85–3.47)	0.13	1.29 (0.59–2.86)	0.52
Duration since HIV diagnosed	≤ 2 years	28/75 (37.3%)	reference		reference	
	> 2years	57/116 (49.1%)	1.62 (0.90–2.93)	0.11	1.36 (0.64–2.87)	0.42
When started on ART	> 90 days before enrollment	59/129 (45.7%)	reference		reference	
	1–90 days before enrollment	9/25 (36.0%)	0.67 (0.27–1.62)	0.37	0.96 (0.34–2.70)	0.94
	At enrollment	17/37 (46.0%)	1.01 (0.48–2.10)	0.98	1.82 (0.72–4.61)	0.21
WHO stage at enrollment	Stage 1	82/181 (45.3%)	reference		reference	
	Stage 2–3	3/10 (30.0%)	0.52 (0.13–2.06)	0.35	0.26 (0.05–1.30)	0.10
IPTp regimen	TMP-SMX alone	49/94 (52.1%)	reference		reference	
	TMP-SMX + DP	36/97 (37.1%)	0.54 (0.30–0.97)	0.04	0.57 (0.31–1.06)	0.07
CD4 T-cell count at delivery	> 500 cells/mm^3^	56/118 (47.5%)	reference		reference	
	250–500 cells/mm^3^	21/57 (36.8%)	0.65 (0.34–1.23)	0.19	0.72 (0.36–1.45)	0.36
	< 250 cells/mm^3^	8/16 (50.0%)	1.11 (0.39–3.15)	0.85	1.91 (0.52–7.00)	0.33
Viral load at delivery	Undetectable	70/145 (48.3%)	reference		reference	
	Detectable	15/46 (32.6%)	0.52 (0.26–1.04)	0.07	0.55 (0.24–1.23)	0.15
Placental malaria	Absent	83/182 (45.6%)	reference		reference	
	Present	2/9 (22.2%)	0.34 (0.07–1.69)	0.19	0.75 (0.12–4.71)	0.76

No significant associations were observed between maternal ACA and formal education, household wealth index, or gestational age at enrollment. Additionally, no significant associations were observed between maternal ACA and the documented HIV disease/treatment characteristics (duration since HIV diagnosis, duration of ART, WHO stage of HIV disease, CD4 T-cell count at delivery, or viral load at delivery).

As shown in Table 3, there were no statistically significant associations between maternal characteristics and the diagnosis of fetal ACA.

**Table 3:** Associations between maternal characteristics and any evidence of fetal ACA.

Characteristic	Category	Risk of any fetal ACA	Univariate		Multivariate	
			OR (95% CI)	p-value	aOR (95% CI)	p-value
Age in years at enrollment	17–21	5/21 (23.8%)	reference		reference	
	22–27	17/53 (32.1%)	1.51 (0.47–4.81)	0.49	1.39 (0.39–4.91)	0.61
	28–43	32/119 (26.9%)	1.18 (0.40–3.48)	0.77	1.59 (0.46–5.47)	0.46
Formal education	Any	45/150 (30.0%)	reference		reference	
	None	9/43 (20.9%)	0.62 (0.27–1.39)	0.25	0.47 (0.19–1.17)	0.11
Household wealth index	Lowest third	18/64 (28.1%)	reference		reference	
	Middle third	17/62 (27.4%)	0.97 (0.44–2.11)	0.93	0.95 (0.41–2.19)	0.90
	Highest third	19/67 (28.4%)	1.01 (0.47–2.17)	0.98	0.89 (0.39–2.06)	0.79
Gravidity	Multigravida	44/152 (29.0%)	reference		Not included in final model due to correlation with maternal age	
	Secundigravida	7/23 (30.4%)	1.07 (0.41–2.79)	0.88		
	Primigravida	3/18 (16.7%)	0.49 (0.14–1.78)	0.28		
Gestational age at enrollment	> 20–28 weeks	21/89 (23.6%)	reference		reference	
	> 16–20 weeks	17/53 (32.1%)	1.53 (0.72–3.26)	0.27	1.75 (0.76–4.02)	0.19
	12–16 weeks	16/51 (31.4%)	1.48 (0.69–3.19)	0.32	2.05 (0.84–5.00)	0.12
Duration since HIV diagnosed	≤ 2 years	25/76 (32.9%)	reference		reference	
	> 2years	29/117 (24.8%)	0.67 (0.36–1.27)	0.22	0.70 (0.32–1.54)	0.38
When started on ART	> 90 days before enrollment	33/130 (25.4%)	reference		reference	
	1–90 days before enrollment	9/25 (36.0%)	1.65 (0.67–4.10)	0.28	1.49 (0.52–4.25)	0.46
	At enrollment	12/38 (31.6%)	1.36 (0.62–2.99)	0.45	1.23 (0.48–3.17)	0.67
WHO stage at enrollment	Stage 1	54/183 (29.5%)	reference		reference	
	Stage 2–3	0/10 (0%)	NA	-	NA	-
IPTp regimen	TMP-SMX alone	31/95 (32.6%)	reference		reference	
	TMP-SMX + DP	23/98 (23.5%)	0.63 (0.34–1.19)	0.16	0.56 (0.28–1.10)	0.09
CD4 T-cell count at delivery	> 500 cells/mm^3^	38/119 (31.9%)	reference		reference	
	250–500 cells/mm^3^	11/58 (19.0%)	0.50 (0.23–1.07)	0.07	0.51 (0.23–1.16)	0.11
	< 250 cells/mm^3^	5/16 (31.3%)	0.97 (0.31–2.98)	0.96	2.05 (0.52–8.10)	0.31
Viral load at delivery	Undetectable	39/147 (26.5%)	reference		reference	
	Detectable	15/46 (32.6%)	1.34 (0.65–2.74)	0.42	1.46 (0.63–3.38)	0.37
Placental malaria	Absent	52/184 (28.3%)	reference		reference	
	Present	2/9 (22.2%)	0.73 (0.15–3.61)	0.70	1.16 (0.18–7.16)	0.87

### Associations between ACA and adverse birth outcomes

After adjusting for maternal age, multivariate analysis showed that women with evidence of severe maternal ACA had a significantly higher risk of PTB (28.6% vs. 6.0%; aOR = 6.04 (1.87–19.5), *p* = 0.003), LBW (33.3% vs. 9.4%; aOR = 4.86 (1.65–14.3), *p* = 0.004), and SGA (28.6% vs. 10.1%; aOR = 3.70 (1.20–11.4), *p* = 0.02) compared to women with no ACA or only mild maternal ACA (Table 4). There was no significant association between evidence of fetal ACA (moderate or severe vs. none/mild) and the risk of adverse birth outcomes.

**Table 4:** Associations between maternal or fetal ACA and adverse birth outcomes.

Location	Intensity	Preterm birth (< 37 weeks)			Low birthweight (< 2500 grams)			Small-for-gestational age		
		Risk of outcome	aOR^a^ (95% CI)	p-value	Risk of outcome	aOR^a^ (95% CI)	p-value	Risk of outcome	aOR^a^ (95% CI)	p-value
Maternal ACA	None-mild	9/149 (6.0%)	reference		14/149 (9.4%)	reference		15/149 (10.1%)	reference	
	Moderate	3/21 (14.3%)	2.43 (0.59–9.99)	0.22	2/21 (9.5%)	1.02 (0.21–4.92)	0.98	1/21 (4.8%)	0.45 (0.05–3.63)	0.45
	Severe	6/21 (28.6%)	6.04 (1.87–19.5)	0.003	7/21 (33.3%)	4.86 (1.65–14.3)	0.004	6/21 (28.6)	3.70 (1.20–11.4)	0.02
Fetal ACA	None-mild	17/173 (9.8%)	reference		23/173 (13.3%)	reference		22/173 (12.7%)	reference	
	Moderate	1/18 (5.6%)	0.50 (0.06–4.05)	0.52	0/18 (0%)	NA	-	0/18 (0%)	NA	-
	Severe	0/2 (0%)	NA	-	0/2 (0%)	NA	-	0/2 (0%)	NA	-

^a^ adjusted for maternal age

## Discussion

### High prevalence of ACA in HIV infected Ugandan cohort

Our study determined the prevalence of ACA in pregnant HIV infected Ugandan women and evaluated for associations between ACA and the risk of three adverse birth outcomes (PTB, LBW, and SGA). The overall prevalence of ACA was 52.8%, maternal ACA was more frequently diagnosed than fetal ACA, and most cases were of mild severity. While these patterns (maternal inflammation more common than fetal inflammation, most cases of mild severity) are consistent with textbook ACA literature drawn from numerous studies in resource rich settings [22], the prevalence we describe is higher than what has been reported for resource rich settings (20% of term deliveries and approximately 50% of preterm deliveries) [23].

Our results help to fill a gap in the literature regarding placental pathology for this understudied patient population. A single prior study in Uganda identified histologic ACA in 34% of 136 placentas delivered at term and evaluated at the Mbarara Regional Referral Hospital [19]. Nearly all women (91.5%) in the Mbarara study were HIV negative. Other notable differences between our analysis and this prior report include our larger cohort that included all deliveries not just those delivered at term gestation, and our microscopic examination was more extensive. Unfortunately, comparing and contrasting different studies is difficult unless identical delivery and diagnostic criteria are utilized. Both the inclusion of pre-term deliveries and the more comprehensive microscopic examination would be expected to show a higher prevalence of ACA and thus this might explain why our study showed such, when compared to the previous Ugandan cohort study. Indeed, a recent study that utilized similar diagnostic criteria to ours documented a similarly high prevalence of histologic ACA in both HIV infected (61.6%) and HIV uninfected (57.1%) Nigerian women [24].

### Strengths and weaknesses

Strengths of this study include the moderately large cohort size, that the diagnosis was rendered by a trained placental pathologist according to the criteria recommended by the Society for Pediatric Pathology [9], and that slides were examined in blinded fashion.

However, we acknowledge certain weaknesses and caveats in both the study design and analysis. All participants resided within a small region of Uganda, thus the findings may not generally represent the entire population. Although our samples size was relatively large compared to previous studies, statistical power was relatively limited to provide precise estimates of measures of association for the socio-demographic and biological risk factors assessed. Furthermore, we only examined a select set of maternal characteristics, which unfortunately did not include several factors associated with the adverse outcomes PTB, LBW, and SGA. Important risk factors for adverse outcomes that were not included in our analysis are maternal stress, prolonged rupture of membranes, duration of labor, previous history of PTB, and medical conditions (such as pre-eclampsia) during pregnancy [25–27]. On the other hand, our study did carefully assess many important maternal characteristics, including HIV disease stage and treatment regimen.

We must also note that our analysis did not include assessment for clinical ACA, nor did we assess for pathogenic causes of ACA (versus “sterile inflammation”) in our samples. Regarding this latter point, while ACA was historically believed to correlate well with microbial infection [22], recent work has in fact suggested that a majority of term deliveries diagnosed with histologic ACA might in fact be “noninfectious” [28]. However, such conclusion must be based upon a truly exhaustive search for pathogens (bacterial, fungal, and viral) and even then it may be impossible to rule out recent past infection that then resolved. Future work with a larger cohort that combines careful histologic and clinical diagnosis of ACA with all known maternal risk factors for both ACA and adverse outcomes should be the next step.

### Maternal risk factors associated with histologic ACA

Often cited maternal risk factors associated with a diagnosis of ACA include nulliparity, younger maternal age, prolonged labor, prolonged rupture of membranes, multiple vaginal exams, internal fetal monitoring, bacterial vaginosis, and Group B streptococcus (GBS) colonization [12, 19, 23, 25, 26, 29]. Unfortunately, our study did not include evaluation for bacterial vaginosis or GBS colonization, nor was there documentation of labor duration and number of vaginal exams. The association between older maternal age and increased risk for ACA identified in our study was surprising because this differs from the established literature. Indeed, textbooks and reviews report an association between younger maternal age and ACA [22, 23]. Interestingly, two recent studies examining risk factors for histologic ACA in preterm and spontaneous term delivery did not find a significant association with young maternal age [29, 30]. Thus, future studies should continue to test for associations between ACA and maternal age, since our findings suggest that different patient populations may show different risk factors.

Intriguingly, we observed a trend towards a reduction in ACA risk for women taking TMP-SMX plus DP compared to TMP-SMX alone. This raises the possibility that malarial chemoprevention regimens might in fact alter the susceptibility to and/or pathogenesis of other infectious agents. Indeed, TMP-SMX has a broad antimicrobial spectrum that includes placental pathogens *Listeria monocytogenes*, *Escherichia coli*, as well as many urinary tract pathogens noted to be associated with increased risk for ACA [31, 32]. To our knowledge, whether the artemisinin-based compound DP, which is effective against malaria [20, 21, 33] and schistosomiasis [34], has antibacterial/fungal properties has not been described, however this may warrant future investigation.

Greater than 600,000 Ugandan women are HIV seropositive [13], and HIV infection and treatment may alter the susceptibility and/or immune response to acute chorioamnionitis. Indeed, numerous reports document increased maternal-to-fetal transmission of HIV in the setting of genital and placental co-infections, including chorioamnionitis [35, 36]. The data from our study show no significant association between maternal HIV disease characteristics and diagnosis of ACA. However, it is important to acknowledge that the majority of women were WHO stage 1 (asymptomatic), with normal CD4 T-cell counts and undetectable viral loads at delivery. Future studies are needed to thoroughly examine whether HIV is a risk factor for ACA.

### Association of maternal ACA with poor birth outcome

Our data showed a significant association between severe maternal ACA and PTB, a finding which is consistent with extensive prior work identifying ACA as a significant risk factor for PTB [7, 8, 12, 17, 37]. There was also an increased risk of LBW and SGA among women with severe maternal ACA, when compared to the combined cohort of women with no evidence of maternal ACA and women with only mild ACA. These data complement those of a recent study evaluating clinical and pathologic characteristics of mothers and placentas from 38 LBW deliveries in Nigeria, where histologic ACA was identified in a high percentage (44%) of such deliveries [38].

There was no significant association between fetal ACA and the risk PTB or LBW. This is somewhat surprising, as fetal ACA is also a well-established risk factor for PTB [7, 8, 12, 17, 37]. The prevalence of PTB in our study (10.2%) was not unusually low, however there were very few cases of severe fetal ACA (only two cases). Previous studies have correlated an association between severe fetal ACA and neurological impairment including cerebral palsy [39, 40]. Our study did not include assessment of such for the newborns.

Finally, we recently reported that placental malaria diagnosis was not associated with a higher risk of adverse birth outcome for the same HIV infected cohort analyzed here for ACA [20]. These results are consistent with that of a prior study in Malawi where ACA but not PM, was found to be associated with a higher risk for PTB [17]. In our current assessment of ACA and risk factors, we did not identify a significant association between ACA and PM. Additional study is required to better define the risk factors for adverse birth outcomes within populations of pregnant of women that are exposed to pathogens not endemic to areas where the bulk of the prior placental pathology literature has focused. Such studies might shed light on interplay among organisms, prophylactic medications, and treatment regimens that alter the biology of preterm and term labor.

### Future investigations

Microbial induced PTB is largely believed to be an inflammatory process where immune mediators disrupt uterine quiescence, culminating in parturition [41, 42]. While delivery of the infected placenta confers a benefit to the mother, sadly, this host defense mechanism comes at the price of prematurity, and all the short and long-term consequences thereof. Studies have found that ACA is mostly polymicrobial in nature, with aerobic and anaerobic commensals from other body sites (gut, vaginal, oral, urinary tract) often implicated [10, 11]. The causative microbes for ACA in resource-limited settings, including Uganda, have yet to be studied. Pre-delivery testing of pregnant woman for GBS carrier status with administration of penicillin to those who test positive is routine clinical practice in the United States, but is not currently part of clinical practice in many resource-limited settings, including our Ugandan population. The potential benefit of such testing and treatment is currently unknown, and thus the incidence of GBS and other placental pathogens within the Ugandan population should be the topic of future investigation. Notably, GBS is the leading cause of neonatal meningitis in Malawi [43], and one report showed microbiologic culture evidence of GBS is some stillbirths associated with ACA in Zimbabwe [44]. Thus, we hope that our current work will serve as the platform for further investigation into the nature of ACA, as well as other placental pathologies, in Uganda and other understudied populations.
